# Effect of kidney-reinforcing and marrow-beneficial traditional Chinese medicine-intervened serum on the proliferation and osteogenic differentiation of bone marrow stromal cells

**DOI:** 10.3892/etm.2014.2062

**Published:** 2014-11-11

**Authors:** DA-AN ZHOU, YUE-NING DENG, LEI LIU, JIAN-JUN LI

**Affiliations:** 1 Department of Spinal and Neural Function Reconstruction, China Rehabilitation Research Center, School of Rehabilitation Medicine of Capital Medical University, Beijing 100068, P.R. China; 2 Department of Rehabilitation, The Third Affiliated Hospital of Liaoning Medical University, Jinzhou, Liaoning 121000, P.R. China

**Keywords:** kidney-reinforcing and marrow-beneficial traditional Chinese Medicine-intervened serum, bone marrow mesenchymal stem cells, osteogenic differentiation capacity

## Abstract

The present study aimed to investigate the effect of kidney-reinforcing and marrow-beneficial traditional Chinese medicine (TCM)-intervened (KRMBTI)-serum on the proliferation and osteogenic differentiation of bone marrow stromal cells (BMSCs) in rats. Rat BMSCs were isolated and cultured *in vitro* with various concentrations of serum obtained from rats at different time-points following treatment with low, medium and high doses of KRMBT. The alkaline phosphatase (ALP) activity and proliferation of the BMCSs was assessed to determine the optimal serum sampling time-point and serum concentration. Transforming growth factor (TGF)-β1 expression of the BMSCs was detected using enzyme-linked immunosorbent assay (ELISA), and hepcidin mRNA expression in the rat livers was detected using reverse transcription polymerase chain reaction. The proliferation of BMCSs treated with serum obtained l h after dosing was observed to be significantly higher than that for BMCSs treated with serum obtained at the four other time-points (P<0.05). Furthermore, the proliferation following treatment with 25% KRMBTI-serum was significantly higher than that for the other KRMBTI-serum concentrations (P<0.01). For a 25% concentration of the serum collected at l h, the proliferation in the high- and low-dose KRMBTI-serum groups was significantly higher than that of the medium-dose and control groups (P<0.01) and no statistical significance was observed between the high- and low-dose groups. In the osteogenic differentiation process of the high-dose group, the ALP activity at every time-point was significantly higher than that of the low-dose group and the peak value of the former was achieved at concentrations between 20 and 30%. KRMBTI-serum was shown to promote the expression of TGF-β1. Furthermore, hepcidin was observed to be expressed at significantly higher levels in the high-dose group than in the control group, and hepcidin expression was significantly higher after 10 weeks compared with that after five weeks. These findings suggest that KRMBTI-serum increases TGF-β1 and hepcidin expression levels, which may be the mechanism underlying the promotion of osteogenic differentiation induced by KRMBTI-serum in BMSCs.

## Introduction

The prevention and treatment of osteoporosis (OP) is a global health problem due to the high morbidity and mortality associated with this condition. Glucocorticoid-induced OP (GIO) may be divided into primary, secondary and idiopathic GIO. Among the types of secondary OP, GIO is the most common. Long-term use of glucocorticoids may impair kidney function, which results in bone damage and loss of myeloid cells. Thus, the pathogenesis of GIO includes kidney asthenia and marrow deficiency (1). Invigorating kidney and nourishing essence Chinese medicine-containing serum has been reported to promote osteogenic differentiation in bone marrow stromal cells (BMSCs) (2,3). Experimental studies have shown that beyond the physiological dose, glucocorticoids promote the differentiation of BMSCs into fat cells and inhibit their differentiation into bone cells (4–6), thereby causing an imbalance in osteoblast-osteoclast coupling, which results in bone loss and GIO. BMSC-derived osteoblasts and adipocytes exhibit intra-plasticity and, under certain conditions, are capable of differentiating into each other. Differentiation among these cells has become a topic of much interest to cytological research (7,8).

The present study aimed to investigate the effects of kidney-reinforcing and marrow-beneficial traditional Chinese medicine (TCM)-intervened (KRMBTI)-serum on rat BMSC proliferation and differentiation *in vitro*. In particular, the effect of KRMBTI-serum on the balance of BMSC osteoblast and adipocyte differentiation and the underlying mechanisms was analyzed.

## Materials and methods

### Animals

A total of 75 pathogen-free, eight-month-old, Sprague-Dawley rats were obtained from the Experimental Animal Center of Liaoning Medical University (Jinzhou, China), 50% of which were male and 50% of which were female. All experimental procedures were performed in accordance with the Guide for the Care and Use of Laboratory Animals of the National Institutes of Health (8th edition, 2011). The animal protocol was reviewed and approved by the Institutional Animal Care and Use Committee of Liaoning Medical University.

### Preparation of reagents

KRMBT was prepared as a suspension containing 10 g lyophilized powder of fresh antler, 5 g oyster powder and 15 g *Epimedium brevicornum* decoction, according to the daily dose required for a 60 kg adult. The osteogenic differentiation-inducing medium was generated by mixing 10–^8^ mol/l dexamethasone, 50 μmol/l vitamin C, 10 mmol/l β-glycerophosphate and 10% fetal bovine serum (FBS) and was stored at 4°C.

### Drug administration

From the group of 75 rats, 45 rats were randomly selected and divided into high-dose (HD), middle-dose (MD) and low-dose (LD) groups (n=15 per group). The rats in each group were randomly divided into five subgroups, with three rats in each subgroup according to the time at which blood was collected. The KRMBT dose was calculated based on the dose/kg body weight of the animal compared with that of humans. The KRMBT doses in the different groups were as follows: LD group, 3.125 g/kg body weight/day; MD group, three-fold that in the LD group; and HD group, nine-fold that in the LD group. KRMBT administration was performed concurrently in all groups for 10 weeks. Thereafter, 5–8 ml abdominal aortic blood was collected at 0.5, 1.0, 1.5, 2.0 and 2.5 h after the last administration at 10 weeks. The blood was then centrifuged at 600 × g for 20 min and the serum was preserved at −70°C.

### Isolation, cultivation and subcultivation of BMSCs

A further 20 rats were dipped in 75% ethanol for 20 min, then anesthetized using an injection of chloral hydrate. Under aseptic conditions, the bilateral lower limb femurs of the rats were separated and the attached fatty, connective tissue and periosteum were removed. Femurs were placed in a small aseptic beaker and transferred to a ultraclean bench. The femurs were then placed in a sterile culture dish. Subsequent to washing with phosphate-buffered saline (PBS), the bilateral ends in the femurs were resected. A total of 5 ml high glucose Dulbecco’s modified Eagle’s medium (DMEM) containing 10% FBS was mixed with 0.5 ml heparin and used to wash the marrow cavity of the femur three or four times. The flushing fluid was fully mixed, followed by cell resuspension with DMEM containing 10% FBS. The cells (1×10^6^ cells/ml) were inoculated in a 25-cm^2^ culture bottle and were incubated at 37°C with 5% CO_2_ and saturated humidity. The medium was changed every three days. After the adherent cells had reached 80–90% confluency, the culture medium was removed and the cells were washed three times with PBS. Preheated (37°C) digestion liquid containing 0.25% trypsin and 0.02% EDTA was used at room temperature to passage the cells at a ratio of 1:2.

### MTT assay

The effects of the different blood sampling time-points, doses of KRMBT and concentrations of KRMBTI-serum on the proliferation of BMSCs were determined by MTT assay. The P3-generation BMSCs were seeded at 1×10^4^ cells/well, and RMBTI-serum was added after 24 h. Five blood sampling time-points (0.5, 1, 1.5, 2, 2.5 h) and 10 KRMBTI-serum addition concentrations (5, 10, 15, 20, 25, 30, 40, 50, 60 and 80%) were set. Each concentration had three repeated wells, and one blank control well was prepared. The final liquid volume of each well was 200 μl. After 72 h, 20 μl 5 mg/ml MTT (Shanghai Sangon Biological Engineering Technology & Services Co., Ltd., Shanghai, China) was added to each well and the plate was incubated for 4 h. Finally the optical density (OD) value at 450 nm of each well was measured using a Synergy NEO HTS plate reader (BioTek Instruments, VT, USA).

### Detection of ALP expression

The P3-generation BMSCs were seeded at a density of 1×10^5^ cells/well, and the serum-free medium was replaced following 24 h of cultivation. After 24 h following the medium replacement, the drug-containing sera at concentrations of 10, 20 and 30% were added for the induction of the BMSCs. Optimum blood sampling time-point, optimum dose and optimum concentration subgroups were established, on the basis of optimum values determined by the MTT assay. The final volume of each well was 1 ml, and a blank control well was also prepared. The supernatant, following induction for 3, 7, 10, 14 and 15 days, was collected. ALP expression was measured by an ELISA method using an ALP kit (R&D Systems Inc., Minneapolis, MN, USA) according to manufacturer’s instructions.

### Detection of TGF-β1 expression

The P3-generation BMSCs with good growth were divided into four groups. These were: the control group, in which the BMSCs were cultured only with culture medium; the osteoblast group, in which BMSCs were cultured with culture medium and osteogenic differentiation-inducing medium; the serum group, in which BMSCs were cultured with culture medium and serum with the optimum concentration for bone differentiation; and the comprehensive induction group, in which BMSCs were cultured with culture medium, osteogenic differentiation-inducing medium and serum with the optimum concentration for bone differentiation. In each group, all the culture medium was replaced every three days. The TGF-β1 concentration in the cell culture supernatant was detected by an ELISA method using a TGF-β1 kit (R&D Systems Inc.) according to manufacturer’s instructions.

### Reverse transcription polymerase chain reaction (RT-PCR) analysis

The remaining 10 rats were randomly divided into control and HD KRMBT groups. In the HD KRMBT group, 3 days after the grouping, KRMBT was intragastrically administered once daily at 10:00 a.m. for 10 consecutive weeks. Experimental samples were taken on the fifth and tenth weeks. The rats were weighed weekly and the dose was adjusted based on changes in body weight. The rats in the control group were administered equal volumes of saline. Bilateral livers were taken 1 h after the final administration, under sterile conditions.

Hepcidin mRNA expression in the liver was determined using an RNA PCR kit (AMV) Ver. 3.0 (Takara Bio Inc., Dalian, China) and a 600 bp DNA ladder marker (Mianyang Gaoxin Tianze Genetic Engineering Co. Ltd., Sichuan, China). Primer Premier 5.0 software (Premier Biosoft, Palo Alto, CA, USA) was used to design PCR primer sequences for β-actin and hepcidin, based on the rat β-actin and hepcidin gene sequences registered in GenBank. Approximately 100 mg fresh rat liver tissue was homogenized in liquid nitrogen. Total RNA extraction was performed using TRIzol reagent (Takara Bio Inc.) according to the manufacturer’s instructions. An RT-PCR kit (Takara Bio Inc.) was used to synthesize the first strand of cDNA according to the manufacturer’s instructions. The RT-PCR reaction conditions were as follows: 42°C for 30 min, 99°C for 5 min, 5°C for 5 min followed by preservation at 4°C in a ABI PCR machine (Applied Biosystems@2720 Thermal Cycler, Grand Island, NY, USA). PCR products were electrophoresed using 3 μl DNA ladder marker with molecular weight standards (100 bp) as the reference. Electrophoresis was performed at 90 V for 1 h.

### Statistical analysis

SPSS software, version 11.0 (SPSS, Inc., Chicago, IL, USA) was used for the statistical analysis of the experimental data. Data are presented as mean ± standard deviation. The electrophoresis results were determined using FluorChem V2.0 software (Model: Gene Genus; Alpha Innotech Corporation, San Leandro, CA, USA). P<0.05 was considered to indicate a statistically significant difference.

## Results

### Optimum BMSC proliferation-promoting conditions

The randomized block-designed analysis of variance (ANOVA) results for the comparison of different blood sampling time-points showed that the OD value of the BMSCs treated with the KRMBTI-serum collected at 1 h was higher than that for the BMSCs treated with KRMBTI-serum collected at the other four time points (P<0.05). Furthermore, the OD value with a 25% concentration of KRMBTI-serum was observed to be significantly higher than that with the other concentrations (P<0.01). Analysis of the different doses using concentrations as covariants showed that P<0.05, indicating that the KRMBT dose exhibited a linear regression correlation with the concentration of KRMBTI-serum added to the BMSCs and the blood-sampling time points (P<0.01; Table I).

**Figure 1:**
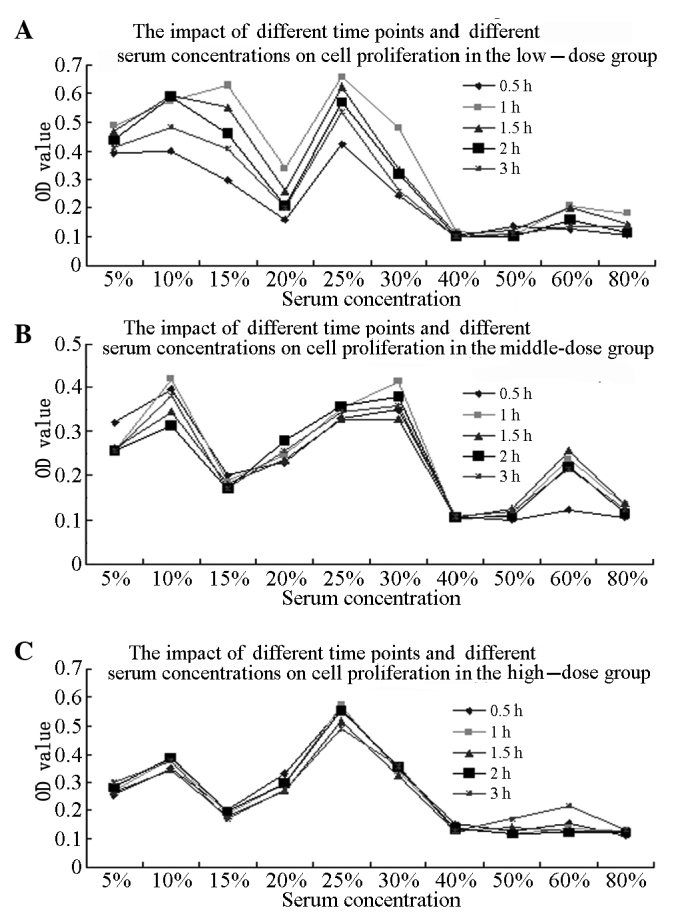
Effect of dose, sampling time-points and serum concentrations on cell proliferation. Effect on cell proliferation in the (A) low-dose, (B) middle-dose and (C) high-dose kidney-reinforcing and marrow-beneficial traditional Chinese medicine-intervened-serum groups at different sampling time-points and added serum concentrations.

ANOVA was performed on the OD values for the HD, MD and LD subgroups at the l-h time-point, with an added concentration of 25%, as well as in the control group. The OD values for the HD and LD subgroups were found to be significantly higher than those for the control and MD groups (P<0.01), but no statistically significant difference was found between the HD and LD groups (P>0.05; Fig. 1).

### Optimum KRMBTI-serum concentration

Analysis of covariance using KRMBTI-serum concentrations as fixed factors revealed that the ALP values in each group at 14 and 15 days were significantly higher than those at 3, 7 and 10 days (P<0.05); however, no significant difference was identified between the HD and LD groups (P>0.05). When induction times were set as fixed factors, ALP values at a concentration of 20% for the LD group and 30% for the HD group were found to be significantly higher than those of other groups (P<0.01), with no significant difference identified between any other two groups (P>0.05). When KRMBTI-serum concentrations and induction times were set as the covariants, the analysis showed that P<0.05, which indicates that the KRMBT dose exhibited a linear regression correlation with the KRMBTI-serum concentration and treatment duration (Table II).

**Figure 2:**
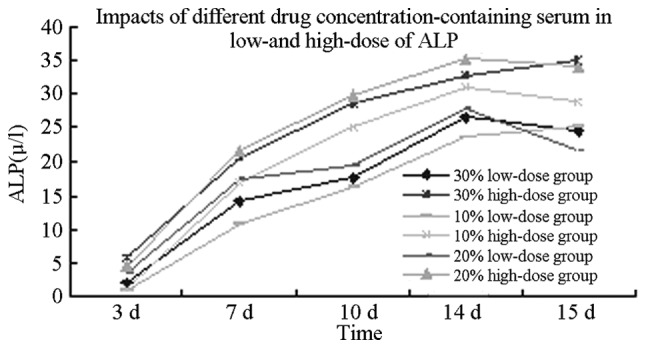
Effect of low and high doses of kidney-reinforcing and marrow-beneficial traditional Chinese medicine-intervened-serum on ALP levels. ALP, alkaline phosphatase.

As shown in Fig. 2, ALP activity in the HD group at the different concentrations and time-points was higher than in the LD group at the corresponding induction time-points. The ALP value peaked between 10 and 14 days when the added concentrations were 20 or 30%, which would significantly promote the osteogenic differentiation of BMSCs.

### TGF-β1 expression

Univariate ANOVA analysis revealed that the TGF-β1 expression levels in the osteoblast, serum and comprehensive induction groups at 14 days were significantly higher than those in the control group (P<0.01). The TGF-β1 expression level in the comprehensive induction group was higher than that in the other groups, with a significant difference among the groups (P<0.01). Furthermore, the TGF-β1 expression level in the osteoblast group was significantly higher than that in the serum group (P<0.05; Table III).

### Hepcidin mRNA expression

**Figure 3:**
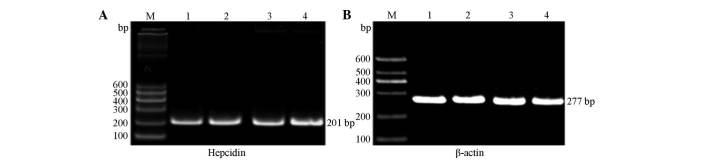
(A) Hepcidin mRNA expression and (B) β-actin in rat kidney tissues detected using reverse transcription polymerase chain reaction analysis. M, DNA Marker DL600; lane 1, normal group, fifth week; lane 2, high-dose group, fifth week; lane 3, normal group, 10th week; and lane 4, high-dose group, 10th week.

**Figure 4:**
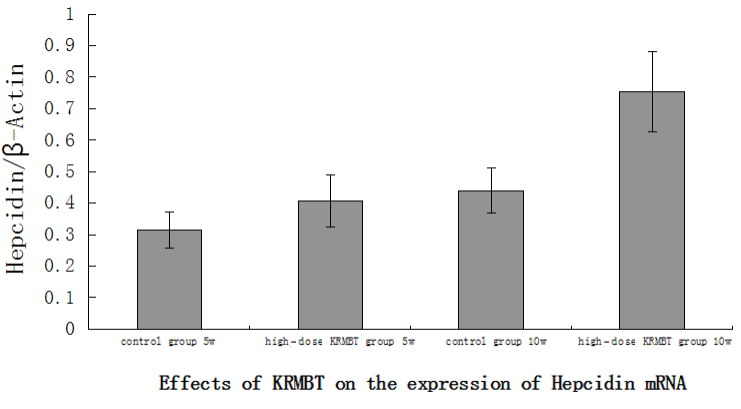
Effect of KRMBT on hepcidin mRNA expression levels. KRMBT, kidney-reinforcing and marrow-beneficial traditional Chinese medicine.

The primer internal reference gene and target genes of each group were subjected to RT-PCR amplification, which was performed using rat liver tissue. RT-PCR analysis revealed two bands at ~201 and 277 bp, as shown in Fig. 3. The image analysis software indicated that the expression of hepcidin in the HD KRMBT group was significantly higher than that in the control group. On the fifth week, hepcidin expression in the HD group was ~1.3-fold higher than that in the control group and on the tenth week it was ~1.8-fold higher (Fig. 4).

## Discussion

A number of scholars believe that supplementing kidney tongbi and strengthening the body to eliminate pathogenic factors are the basic principles in the treatment of bone osteoporosis (9).

Oysters are naturally salty and are high in natural vitamin D-like substances, iron and zinc, all of which enter the kidneys (10,11). The CHCl_3_ extract of deer antler has been reported to inhibit the activity of differentiated osteoclasts and to regulate bone resorption (12). The aqueous extract, deer antler aqua-acupuncture (DAA), is rich in antioxidant polyphenols, which have been shown to regulate bone circulation and treat arthritis (13). Furthermore, *E. brevicornum* has been found to promote the activity of osteoblasts and to inhibit the differentiation of osteoclasts, as well as to promote the osteoblast differentiation of BMSCs (14,15). Qian *et al* (12) reported that *E. brevicornum* regulates core binding factor α1 expression. Icariside I and II and epimedin B and C, which may be extracted from Epimedii Brevicornus, have also been reported to promote *in vitro* osteoblast proliferation and mineralization (15,16).

In the present study, various KRMBT groups, including three dose groups and five blood sampling time-points with final concentrations between 0 and 80% were investigated. A dose-effect relationship was observed and the optimum BMSC proliferation-promoting effect was found for serum sampled 1 h after the administration of KRMBT, while the optimum KRMBTI-serum concentration was observed to be 25%. These findings are consistent with those of a previous study (17). The optimum BMSC osteogenic differentiation-promoting serum concentration was between 20 and 30% in the HD group.

TGF-β is one of the most abundant cell growth factors present in bone tissues. This factor not only promotes osteoblast proliferation and differentiation, but also stimulates bone formation, supports osteoclast formation and stimulates bone resorption. TGF-β is an important coupling factor in the regulation of bone formation and resorption (18,19). In addition, TGF-β has an important function in inducing bone and cartilage formation and calcification in different periods of fracture healing (20). Shin *et al* (21) and Ozmen *et al* (22) demonstrated that Erk channels are involved in the osteogenic differentiation of BMSCs, and that TGF-β1 activates such channels. In the present study, KRMBT was found to promote TGF-β1 expression.

Houtkooper *et al* (23) reported that lactoferrin promotes the proliferation of osteoblasts and inhibits the activity of osteoclasts *in vivo*. Moreover, when the calcium intake of postmenopausal women is sufficient, iron intake levels have been found to be positively correlated with bone mineral density (23). Hepcidin, an ion regulator isolated from human urine and named by Park *et al* (24) in 2001, has been reported to regulate the absorption, utilization, storage and re-use of the iron alternative pathway. Pigeon *et a*l (25) confirmed that increased dietary iron content or increased hepatic iron may increase liver hepcidin expression. In the present study, hepcidin expression in the HD KRMBT group was observed to be significantly higher than that in the control group and was significantly higher on the tenth week compared with the fifth week, indicating that KRMBT increased hepcidin expression. This mechanism may be the underlying process by which KRMBT treats osteoporosis.

## Figures and Tables

**Table I:** Effect of dose, serum sampling time-point and concentration of kidney-reinforcing and marrow-beneficial traditional Chinese medicine-intervened-serum on bone marrow stromal cell proliferation as determined by MTT assay.

	Optical density				
	
Parameters	0.5 h	1.0 h	1.5 h	2.0 h	2.5 h
Low-dose group (%)					
0	0.2354±0.0436	0.2424±0.0357	0.2652±0.0463	0.2571±0.0642	0.2523±0.0384
5	0.3932±0.0876	0.4869±0.1540	0.4668±0.0378	0.4370±0.0768	0.4094±0.0154
10	0.4099±0.0557	0.5732±0.0162	0.5954±0.0852	0.5902±0.0159	0.4849±0.0198
15	0.2954±0.0286	0.6281±0.0318	0.5523±0.0428	0.4589±0.0078	0.4071±0.0077
20	0.1609±0.0390	0.3391±0.0396	0.2698±0.0843	0.2104±0.0193	0.2024±0.0542
25	0.4209±0.0874	0.6583±0.1231^a^	0.6256±0.1678	0.5714±0.1072	0.5363±0.1347
30	0.2423±0.0546	0.4813±0.0398	0.3349±0.0430	0.3182±0.0251	0.2569±0.0279
40	0.1034±0.0152	0.1189±0.0295	0.1123±0.0239	0.1023±0.0218	0.1029±0.0364
50	0.1379±0.0542	0.1073±0.0145	0.1209±0.0146	0.1017±0.0255	0.1089±0.0137
60	0.1251±0.0126	0.2079±0.0518	0.2023±0.0198	0.1609±0.0355	0.1384±0.0148
80	0.1079±0.0281	0.1807±0.0325	0.1459±0.0145	0.1149±0.0335	0.1384±0.0289
Middle-dose group (%)					
5	0.3209±0.0257	0.2574±0.0385	0.2609±0.0529	0.2573±0.0392	0.2582±0.1389
10	0.3970±0.0333	0.4189±0.0546	0.3459±0.0534	0.3125±0.0535	0.3809±0.0169
15	0.2009±0.0187	0.1849±0.0236	0.1814±0.0436	0.1608±0.0171	0.1703±0.0122
20	0.2310±0.0354	0.2459±0.0123	0.2351±0.0252	0.2799±0.0255	0.2580±0.0353
25	0.3289±0.0678	0.3522±0.0117	0.3279±0.0557	0.3583±0.0254	0.3462±0.0327
30	0.3489±0.0242	0.4129±0.0574	0.3284±0.0336	0.3783±0.0534	0.3592±0.0247
40	0.1007±0.0152	0.1013±0.0067	0.1049±0.0365	0.1059±0.0384	0.1068±0.0188
50	0.1012±0.0033	0.1119±0.0143	0.1231±0.0130	0.1082±0.0165	0.1209±0.0274
60	0.1223±0.0160	0.2379±0.0825	0.2569±0.1686	0.02219±0.0401	0.2164±0.0209
80	0.1050±0.0022	0.1348±0.0294	0.1359±0.0203	0.1161±0.0567	0.1253±0.0152
High-dose group (%)					
5	0.2542±0.0487	0.2709±0.0182	0.2682±0.0388	0.2802±0.0394	0.3019±0.2661
10	0.3501±0.0356	0.3831±0.0534	0.3479±0.0561	0.3892±0.0716	0.3775±0.0239
15	0.2029±0.0126	0.1902±0.0336	0.1839±0.0465	0.1969±0.0151	0.1713±0.0157
20	0.3314±0.0264	0.2969±0.0178	0.2704±0.0357	0.2981±0.0479	0.2729±0.0345
25	0.5634±0.0678	0.5723±0.0234^a^	0.5164±0.0688	0.5569±0.0769	0.4912±0.0634
30	0.3413±0.0377	0.3369±0.0568	0.3235±0.0367	0.3524±0.0367	0.3583±0.0257
40	0.1507±0.0147	0.1359±0.0137	0.1309±0.0344	0.1359±0.0474	0.1284±0.0342
50	0.1249±0.0130	0.1189±0.0243	0.1479±0.0468	0.1192±0.0321	0.1709±0.0321
60	0.1573±0.0278	0.1372±0.0587	0.1289±0.2334	0.1224±0.1335	0.2159±0.0178
80	0.1109±0.0121	0.1289±0.0322	0.1274±0.0243	0.1208±0.0467	0.1321±0.0243

Data are presented as the mean ± standard deviation of the optical density values at 450 nm.

a P<0.01 vs. the control (0%) group.

**Table II:** Effect of dose, induction time and concentration of kidney-reinforcing and marrow-beneficial traditional Chinese medicine-intervened-serum on bone marrow stromal cell differentiation.

	Alkaline phosphatase (U/l)				
	
Parameters	3 days	7 days	10 days	14 days	15 days
Low-dose group (%)					
0	4.8975±0.0586^a^,^b^	4.7546±0.0346^a^,^b^	4.4281±0.3449^a^,^b^	4.6847±0.0487^a^,^b^	4.1469±0.0254^a^,^b^
10	2.1063±1.2771^a^,^b^	14.1718±1.2270^a^,^b^	17.6483±1.5439^a^,^b^	26.6462±3.3789^a^,^b^	24.6012±1.2270^a^,^b^
20	5.7873±0.9371	20.7154±1.4168	28.4867±2.3227	32.7812±2.7664	35.0307±1.6232
30	1.0839±1.2771^a^,^b^	10.4061±1.2032^a^,^b^	15.9518±0.5355^a^,^b^	23.5787±2.1545^a^,^b^	25.2147±1.2270^a^,^b^
High-dose group (%)					
10	1.2883±0.6135^a^,^b^	16.8302±2.1545^a^,^b^	25.0102±2.3227^a^,^b^	31.1452±2.1545^a^,^b^	28.8957±2.4540^a^,^b^
20	3.0538±0.9371^a^,^b^	17.4438±1.8743^a^,^b^	19.4888±1.8657^a^,^b^	27.8732±1.7986^a^,^b^	21.7381±1.8513^a^,^b^
30	4.3558±1.2270	21.7659±0.9375	29.7136±2.1546	35.2352±0.9482	34.0082±0.9482

Data are presented as the mean ± standard deviation.

a P<0.01 vs. low-dose group in 20% dosage;

b P<0.01 vs. high-dose group in 30% dosage.

**Table III:** TGF-β1 expression in bone marrow stromal cells in the different treatment groups.

Group	TGF-β1 (pg/ml)
Control	15.3992±3.1623
Osteoblast	41.7784±10.8950^a^,^c^
Serum	31.7868±2.0429^a^
Comprehensive induction	69.8832±4.3906^a^,^b^,^d^

Data are presented as the mean ± standard deviation; n=5 per group.

a P<0.01 vs. the control group;

b P<0.01 vs. the osteoblast group;

c P<0.05,

d P<0.01 vs. the serum group.

TGF, transforming growth factor.
